# Detraining Effects on Muscle Quality in Older Men with Osteosarcopenia. Follow-Up of the Randomized Controlled Franconian Osteopenia and Sarcopenia Trial (FrOST)

**DOI:** 10.3390/nu13051528

**Published:** 2021-05-01

**Authors:** Mansour Ghasemikaram, Klaus Engelke, Matthias Kohl, Simon von Stengel, Wolfgang Kemmler

**Affiliations:** 1 Institute of Medical Physics, Friedrich-Alexander University of Erlangen-Nürnberg, 91054 Erlangen, Germany; simon.von.stengel@imp.uni-erlangen.de (S.v.S.); Wolfgang.Kemmler@imp.uni-erlangen.de (W.K.); 2 Department of Medicine III, Friedrich-Alexander University of Erlangen-Nürnberg, University Hospital Erlangen, Ulmenweg 18, 91054 Erlangen, Germany; klaus.engelke@imp.uni-erlangen.de; 3 Faculty Medical and Life Sciences, University of Furtwangen, 78054 Villingen-Schwenningen, Germany; kohl@hs-furtwangen.de

**Keywords:** exercise training, detraining, resistance training, muscle mass, muscle strength

## Abstract

The present study aimed to determine the effect of detraining on muscle quality (MQ) in older men with osteosarcopenia. Forty-three community-dwelling older men (78 ± 4 years) were randomly allocated to a consistently supervised high-intensity resistance exercise training (HIRT) group (*n* = 21) or a control group (CG, *n* = 22). The HIRT scheduled a periodized single set protocol twice weekly. After the intervention, the men were subjected to six months of detraining. Muscle quality (MQ), defined as maximum isokinetic hip/leg extensor strength per unit of mid-thigh intra-fascia volume, was determined by magnetic resonance imaging (MRI) or per unit of thigh muscle mass assessed by dual-energy X-ray absorptiometry (DXA). Intention-to-treat analysis with multiple imputations was applied. We observed significant exercise effects for MQ (*p* = 0.001). During detraining, the HIRT group lost about one-third of the intervention-induced gain and displayed significantly (*p* = 0.001) higher MQ reductions compared to the CG. Nevertheless, after training and detraining, the overall intervention effect on MQ remained significant (*p* ≤ 0.004). In summary, six months of absence from HIRT induce a significant deleterious effect on MQ in older osteosarcopenic men. We conclude that intermitted training programs with training breaks of six months and longer should be replaced by largely continuous exercise programs, at least when addressing MQ parameters.

## 1. Introduction

Losing muscle mass and function (i.e., sarcopenia) are undeniable developments during advanced aging. Recent evidence suggests that dynamic resistance exercise training, be it with or without dietary supplementation (e.g., [1,2]), might be the most effective agent to affect age-related sarcopenia [3]. However, considering that breaks in exercise routines might be frequent situations in older adults’ lives [4], it is surprising that only a few studies address “detraining” on sarcopenia parameters after previous conditioning. Further, when focusing on moderate-long training breaks (3–6 months), i.e., a scenario brought about by diseases, injuries or the present COVID-19 pandemic, the results of current studies are inconsistent, ranging from complete loss [5,6] to partial preservation of strength gains [7,8]. There is some evidence that muscle mass is more vulnerable to detraining effects than neuromuscular effects [5,6]. Indeed, in a recent detraining study in older men [9], we reported significant reductions in both muscle mass and function, with much more pronounced effects on mass. Considering the relevance of both parameters in the field of sarcopenia [10], it may be a good idea to focus on muscle quality (MQ), typically defined as muscle strength per unit of muscle mass [11], as a combined study outcome in training or detraining studies. In the present study, we particularly aimed to determine the effect of detraining on MQ. However, we applied both dual-energy X-ray absorptiometry (DXA) as the reference standard to determine lean body/muscle mass [12] and the advanced magnetic resonance imaging (MRI) technique to provide reliable results.

Our primary hypothesis was that 6 months of detraining after 18 months of dynamic resistance exercise (HIRT) significantly decrease MQ defined as maximum hip/leg extensor muscle strength adjusted to intra-fascia volume (primary outcome) of the mid-thigh (MRI) or to lean body mass (LBM) of the thigh (DXA) compared to a non-training control group. We also hypothesize (secondary hypothesis) that the overall effects on MQ after training and detraining in older men with osteosarcopenia, i.e., the simultaneous presence of osteopenia and sarcopenia defined as reduced bone [13] and muscle mass [14,15], still remained significant.

## 2. Methods

This randomized controlled trial was performed by the Institute of Medical Physics, Friedrich-Alexander University of Erlangen-Nürnberg, Germany, between February 2018 and June 2020. The study was registered under ClinicalTrials.gov (accessed on 5 November 2020): NCT03453463 (training) and NCT04444661 (detraining). The investigation fully complied with the Helsinki Declaration, and all methods were approved by the university ethics committee (numbers 67_15b and 4464b, 4464b amendment) and the federal bureau of radiation protection (BfS, number Z 5-2246212-2017-002). Written informed consent was obtained from all study participants after they received detailed information. Of importance, the detraining approach of FrOST was not a preplanned study but predominately generated by the COVID-19 pandemic.

### 2.1. Participants

**Figure 1:**
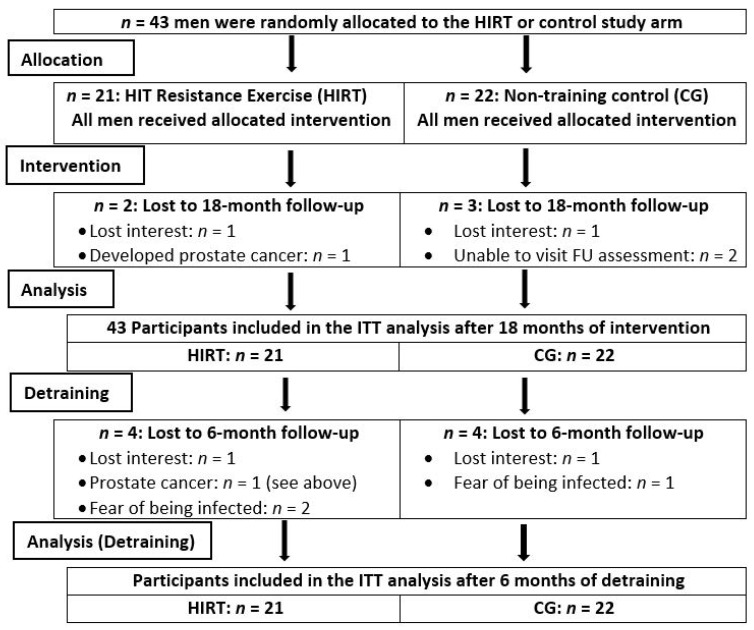
Flowchart of the FrOST study. CG, control group; HIRT, high-intensity resistance exercise training.

The recruitment process has already been published in detail [9,16]. Briefly, inclusion criteria were (a) morphometric sarcopenia (skeletal muscle mass index (SMI) ≤ 7.26 kg/m^2^ [14,15]) and (b) osteopenia or osteoporosis at the lumbar spine (LS) or total hip (tHip) [17]), while exclusion criteria were (a) secondary osteoporosis, (b) skeletal muscle disease or treatment known to affect muscle and bone during the last 2 years, (c) hip fractures, (d) any limitations to intensive exercise training, (e) experience in resistance training during last two years, and (f) alcohol consumption > 60 g/d ethanol. Finally, 43 eligible men (72 years and older) were randomly allocated to an exercise (EG, *n* = 21) or control (CG, *n* = 22) group (Figure 1).

### 2.2. Randomization and Blinding Procedures

Randomization and blinding procedures have been extensively described in previous publications (e.g., [9,16]). Briefly, participants allocated themselves to the HIRT or control group by drawing lots. Neither participants nor researcher knew the allocation beforehand (allocation concealment). Blinding refers exclusively to the test/outcome assessors, who were kept unaware of the participants’ group status (HIRT or CG).

### 2.3. Training and Detraining

Details of the exercise have been frequently published (e.g., [9,16]). Briefly, we applied a progressive, periodized and consistently supervised HIRT, defined as a single set exercise protocol to repetition maximum [18] using intensifying strategies [19]. Participants consistently exercised two times/week on machines (MedX, Ocala, FL, USA) in a centrally located gym (Kieser Training, Erlangen, Germany). In cases of temporary inability (holidays, illness), they were allowed to exercise three times in the week before and/or after. Twelve to fourteen exercises/session taken from a pool of 18 exercises (latissimus front pulleys, pull-overs, seated rowing, back extension, inverse fly, bench press, military press, lateral raises, butterflies, crunches, lateral crunches, calf raises, leg press, leg extension, leg curls, leg adduction, leg abduction, hip extension,) were conducted. Exercise intensity was prescribed by a given range of repetitions (reps)/set (i.e., 5–7) and the corresponding set endpoint [18] (“effort”) specified as “non-repetition maximum” (nRM), “self-determined repetition maximum (RM) and “work to momentary failure” (MF). The 18 month HIT-RT was structured into eight periods of 8–12 weeks with progressively increasing intensity/effort using intensifying strategies [19]. Apart from the initial familiarization and conditioning phase 1, each period was organized in linearly periodized mesocycles of 4 weeks, with each 4th week as a low-intensity/regeneration week. Relative intensity (i.e., % of repetition maximum,% RM) during the 4 weeks linearly periodized mesocycles ranged between 65 and 85% 1RM; about 40% of the sessions prescribed an explosive concentric movement during the exercise.

Due to the situation that detraining was not a preplanned part of the FrOST project, we aimed to include participants in our university health sports club and continue their exercise routine after study end (December 2019). However, due to holidays, logistic issues and finally, the COVID-19 related lockdown of all training facilities in Bavaria, we could not consider restarting exercise training until mid-June 2020. Given this situation, none of the participants conducted any resistance training during the detraining period from mid-December 2019 and mid-June 2020 (i.e., 6 months). However, as people were allowed to conduct individual outdoor activities, apart from HIRT, the physical activity of most participants remained quite constant.

### 2.4. Supplements

Dietary protein intake was assessed at baseline throughout 4 day dietary protocols (Freiburger nutrition record, Nutri-Science, Hausach, Germany). All participants were provided with supplementation of whey protein (Active PRO80, inkospor, Roth, Germany; HIRT: 1.5–1.7 g vs. CG: 1.2–1.3 g/kg/d), cholecalciferol (MYVITAMINS, Manchester, UK) and calcium (Sankt Bernhard, Bad Dietzenbach, Germany) during the 18 month training phase. While protein and calcium supplementation was terminated at intervention end (December 2019), due to the non-preplanned detraining approach, all subjects were provided with cholecalciferol up to June 2020 as a way of thanking them for their compliance during the intervention and/or tests.

### 2.5. Study Outcomes

The FrOST follow-up data as determined after 6 months [19], 12 months [20] and 18 months [21] on primary and secondary study outcomes, as well as corresponding general detraining data [9], were published in earlier contributions. As stated, this contribution focused on detraining effects on muscle quality (MQ).

#### 2.5.1. Primary Study Outcome

- Muscle quality, defined as maximum hip/leg extensor strength (MILES) per unit of mid-thigh intra-fascia volume as determined by MRI during detraining.

#### 2.5.2. Secondary Study Outcome

- Muscle quality, defined as MILES per unit of thigh muscle mass as determined by DXA during detraining.

### 2.6. Assessments

Assessments that were predominately related to the intervention (e.g., 4 day dietary protocol, calcium questionnaire) have been reported in detail in previous publications.

Participants completed a standardized questionnaire that asked for (a) diseases, operations, drugs, and supplements, (b) demographic parameters, (c) physical disabilities, (d) stumbles and injurious falls, (e) recent low-trauma fractures, and (f) daily life activities, including exercise and physical activity [22,23]. The follow-up questionnaires focus on potential changes that may affect our study results. In particular, emphasis was placed on detecting changes in exercise and physical activity during the detraining period. To adequately evaluate these parameters, we used our physical activity and exercise questionnaire [22,23], particularly developed to determine mechanical loading. Briefly, we calculated two activity and two exercise indices from the questionnaires. The overall physical activity score summarizes household, hobby, gardening activities and occupational activities indoor and outdoor. The weight-bearing (not given) physical activity index includes weight-bearing activities only. In parallel, the overall exercise index (min/week) characterizes weekly exercise frequency and duration (Table 1), while the osteogenic exercise index (not given) (The corresponding indices considered the osteopenia aspect of the study.) The osteogenic index (min/week) additionally considers the osteogenic relevant intensity of the specific exercise discipline.

All participants were requested to record three weekdays and one weekend day characteristic for their dietary habits at baseline, 6, 12, 18 and 24 months follow-up (FU). Participants were provided with simple diet records (Freiburger nutrition record, Nutri-Science, Hausach, Germany).

For consistency, completeness and accuracy, the questionnaires were carefully checked. All assessments were highly standardized. The same research assistant consistently led, supervised and/or analyzed a given assessment at about the same time of day (±2 h). Furthermore, tests were consistently conducted with the same calibrated devices, in identical order, at the same location.

### 2.7. Isokinetic Strength Testing

MILES was measured using an isokinetic leg press (CON-TREX LP, Physiomed, Laipersdorf, Germany). The participants sat in an adjustable chair with support for the back, slightly supine (15°) position, fixed by hip and chest straps. Using the standard velocity of 0.5 m/s, the range of motion within the knee angle was 30–90°. After briefing and familiarization, the participants conducted five repetitions with maximum voluntary effort (“push as strongly as possible”). The higher value of two trials intermitted by 2 min of rest was included in the analysis. The coefficient of variation (CV) and intraclass correlation coefficient (ICC) for maximum bilateral isokinetic hip-/leg-extension strength in this cohort was 0.990 (ICC) and 4.4% (CV).

### 2.8. Body Composition and Muscle Quality

Body height and mass were determined using calibrated devices. Body composition and thigh LBM were evaluated by DXA (QDR 4500a, Discovery-upgrade, Hologic Inc., Bedford, MA, USA), according to the manufacturer’s specification. The thigh region was segmented from the lower end of the os ischia to the lateral knee joint cavity. MRI acquisition was performed using a 3 T scanner (MAGNETOM PRISMA^fit^, Siemens Healthineers AG, Erlangen, Germany) and an 18-channel body surface coil. Image analysis was conducted using the medical image analysis framework (MIAF), FAU. Segmentation was based on a multi-stage approach combining fuzzy c-means clustering, level sets and 3D filtering of structures belonging to the fascia lata as described previously [24].

MILES (N) per unit of thigh LBM (DXA) or intra-fascia volume (MRI) was used to calculate muscle quality (DXA: N/g; MRI: N/cm^3^).

### 2.9. Statistical Analysis

Intention to treat analysis with multiple imputations (ITT) using R statistics software (R Development Core Team Vienna, Austria) in combination with Amelia II [25] was applied to address our research question. The full data set was used for ITT, and imputation repeated 100 times, which worked well in all cases. After checking the normal distribution of the data, all the study outcomes addressed here were analyzed by dependent t-tests, applying t-test comparisons with pooled SD. To compare differences for changes during detraining (i.e., time–group interactions = effects) between the HIRT and the CG, we applied an ANCOVA that adjusted for baseline (i.e., pre-intervention) and 18 month differences (i.e., baseline data of the detraining research issue) of the corresponding parameter. All tests were 2-tailed, significance was accepted at *p* < 0.05.

## 3. Results

Figure 1 displays the participant flow through the study. In summary, two participants, who lost interest and the participant with prostate cancer, dropped out during the intervention phase. Two other participants of the CG were unable to visit the 18 month follow-up assessment. During the detraining period, three participants did not take up our invitation to the six-month detraining follow-up due to fear of being infected with COVID-19.

Participant characteristics after 18 months of intervention, i.e., baseline characteristics for the detraining period, are listed in Table 1. Of importance, data did not vary significantly between the groups.

### 3.1. Study Outcomes

During the intervention period, we observed significant effects (*p* < 0.001) for MQ defined as “MILES/intra-fascia volume” (Table 2). During detraining, MQ decreased significantly (*p* < 0.001) in the HIRT and increased non-significantly in the CG. Detraining changes were significantly higher in the HIRT compared with the CG (*p* < 0.001). However, after 18 months of training and 6 months of detraining, we still observed significantly more favorable changes in MQ in the HIRT compared to the CG (*p* = 0.004) (Table 2). Thus, we confirmed our primary and secondary hypotheses for the primary study outcome.

Table 2 displays the data for secondary study outcomes. Largely in parallel to MILES/intra-fascia volume, MQ defined as MILES/thigh LBM increased significantly (*p* < 0.001) in the HIRT and remained largely stable in the CG (*p* = 0.771) during the intervention period. Corresponding differences between the groups were significant (*p* ≤ 0.001). During the detraining period, MILES/thigh LBM decreased significantly in the HIRT (*p* = 0.004) and remained stable in the CG (*p* = 0.824). The corresponding detraining effect was significant (*p* = 0.001). Nevertheless, after training and detraining, we still observed a significant intervention effect (*p* ≤ 0.001) for MILES /thigh LBM (DXA). Thus, we also confirmed our hypothesis that 6 months of detraining after 18 months of HIRT significantly decrease MQ as assessed by DXA compared to a non-training control group, but that the overall effects on MQ after training and detraining remained significant.

### 3.2. Confounding Parameters

Most importantly, during the six-month FU period, none of the participants conducted a supervised group exercise or resistance-type exercise training. Total exercise volume during the detraining phase (i.e., from intervention end to six-month FU) increased slightly in the CG (*p* = 0.778) and decreased significantly (*p* < 0.001) in the EG. Of importance, both groups increased the number of outdoor activities (20 ± 29%, *p* ≥ 0.103) with an aerobic character (particularly brisk walking and biking). According to the physical activity and exercise questionnaire, habitual physical activity did not vary significantly in the EG and CG over the entire 24 month period. Correspondingly, habitual physical activity during the detraining period did not differ (*p* < 0.442) from data determined for the EG and CG at baseline or after 6, 12 and 18 months. In parallel, dietary calcium and protein intake (Table 1) did not change relevantly during detraining. As cholecalciferol supplementation was maintained during the detraining period, 25-OHD levels increased slightly (3–5%) in both groups. Relevant injuries, operations, diseases, changes of pharmacologic therapy or periods of physical inactivity >1 week were not recorded during the detraining period.

## 4. Discussion

Particularly in the vulnerable cohort of sarcopenic people, temporal discontinuation of exercise may have serious consequences on “MQ”, defined as maximum strength per unit of muscle mass. The present project is the first randomized controlled trial to report MQ response after training and detraining in men with osteosarcopenia. In summary, apart from the anticipated exercise effect of HIRT on MQ, we confirmed our hypotheses (1) that MQ decreased supra-normally, i.e., significantly more pronounced compared to an untrained CG, during detraining, while (2) the overall effect of exercise on MQ remained significant after six months of detraining.

Only a few other studies have reported MQ values after detraining. Unfortunately, their short training periods [6,26] prevent a meaningful comparison with our result. Correa et al. [26] applied 12 weeks of progressive strength training followed by a 12 week period of detraining in elderly women. In contrast to our study, MQ (Maximum leg extensor strength per unit of rectus femoris volume as determined by ultrasound) of the exercise group (a CG was not implemented) demonstrated a linear, albeit non-significant, increase during the training and detraining period. Of surprise and potentially causal for this finding, the authors reported similar pronounced changes of the underlying parameters “rectus femoris muscle volume” and “knee extensor 1RM” after training (Δ 25–32%, *p* ≤ 0.002) and detraining (Δ 19–21%, *p* ≤ 0.042).

In another study, Ivey et al. [6] studied the effects of age and gender on MQ (Maximum leg extensor strength per unit of quadriceps muscle volume as determined by MRI) responses to 9 weeks of high-volume heavy resistance training and 31 weeks of detraining. Concerning their older male cohort, the authors reported significant increases (≈15%) in MQ after training and a reduction of about one-third of the exercise-induced gain during the 31 weeks of detraining (FrOST: 38% reduction (MRI-based) of the exercise-induced gain after 26 weeks of detraining). However, MQ remained significantly above baseline levels. Although both findings correspond with our data, it is amazing that differences in intervention length (9 weeks vs. 18 months) did not affect the extent of detraining effects. Considering the principle of “adaptation stability” [27], one would have expected adaptation achieved over a longer period to be characterized by higher stability. Indeed, there is some evidence that the capacity to sustain training-induced hypertrophy in elderly people is related to the intensity, volume, and duration of the training protocol [5]. Fatouros et al. [28] emphasize the relevance of exercise intensity in this context while demonstrating that high-intensity (80–85% 1RM) RT protocols are superior to low-intensity (50–55% 1-RM) one for preserving muscle strength during 4 months of detraining.

Revisiting our result on MQ in detail, we observed a much more pronounced increase in MILES than muscle mass (or volume) during the HIRT [16,29]. On the other hand, the relative decline of muscle mass (vs. MILES) was much more pronounced during detraining [9]. The latter finding confirmed the observation [5,6] that neuromuscular effects, which contribute to strength and power, are more resistant to detraining compared with hypertrophic effects, resulting in higher preservation of strength compared to muscle mass/size gains during detraining (e.g., [26,30]).

Some study limitations and features should be considered. First of all, we did not apply a preplanned (RT) detraining approach but used the temporary COVID-19 related ban on supervised indoor activities to evaluate detraining effects on MQ. Of importance, detraining refers exclusively to the absence of RT exercise, while aerobic-type exercise increased slightly during the detraining period, a situation that differs from other studies (e.g., [28,31,32]). Related to our somewhat accidental approach, we provided all participants with cholecalciferol supplements post-intervention as an appreciation for their dedicated and reliable participation. Further, it might have been more meaningful to focus on shorter detraining periods, which tend to be more common in people’s exercise patterns (e.g., three months). However, due to the lockdown of university facilities, we were unable to conduct the assessment earlier. Another potential limitation was the lack of quantitative Dixon images due to a technical error of the MRI scanner. These data would have allowed us to measure changes in muscle fat infiltration.

In summary, six months of absence from RT lead to deleterious effect on MQ in older men with osteosarcopenia, even the HIRT intervention was of long duration, and habitual physical activity was not limited. As a consequence, the intermitted training programs of many healthcare providers with six months of training breaks should be replaced by largely continuous exercise programs, at least when addressing MQ parameters.

## Figures and Tables

**Table 1:** Baseline characteristics (i.e., before detraining) of the participants of the exercise and control group. CG: control group; EG: exercise group.

Variable	CG (*n* = 22) MV ± SD	HIRT (*n* = 21) MV ± SD
Age (years)	80.8 ± 4.7	79.6 ± 3.6
Body mass index (kg/m^2^)	24.6 ± 2.1	24.8 ± 3.0
Total body fat (%)	32.2 ± 5.5	33.5± 4.3
More than two diseases (*n*)	13	9
Metabolic syndrome (*n*) ^a^	12	9
Diabetes mellitus type II (*n*)	1	1
Habitual physical activity (Index) ^b^	4.32 ± 1.44	4.51 ± 1.27
Exercise volume (min/week)	54 ± 56	52 ± 50
25-OHD level (ng/mL) ^c^	29.6 ± 5.8	28.1 ± 5.8
Calcium intake (mg/d)	852 ± 266	827 ± 291
Energy intake (kcal/d) ^d^	2242 ± 639	2197 ± 508
Dietary protein intake (g/kg/d) ^d^	1.25 ± 0.23	1.15 ± 0.27

^a^ according to the International Diabetes Federation; ^b^ scale from (1) “very low” to (7) “very high”; ^c^ 25-hydroxy-vitamin D, ECLIA; Roche Diagnostics, Penzberg, Germany; ^d^ as determined by a 4 day dietary record.

**Table 2:** Baseline values and mean changes ± standard error (SE) of muscle quality (MILES/intra-fascia volume and MILES/thigh mass) as determined by MRI and DXA in the HIRT and CG after training and detraining.

		Baseline (SE)	Δ Training ^a^ (SE)	Δ Detraining ^b^ (SE)	Δ Overall ^a^ (SE)
MILES/intra-fascia volume (N/cm^3^)	CG	1.55 (0.07)	0.01 (0.05)	0.04 (0.04)	0.05 (0.04)
	HIRT	1.47 (0.11)	0.49 (0.05)	−0.18 (0.05)	0.30 (0.05)
	*p*	0.54	0.001	**0.001**	**0.004**
MILES/thigh LBM (N/kg)	CG	195 (10)	1.8 (4.8)	0.5 (3.2)	2.2 (2.9)
	HIRT	176 (13)	50.5 (4.6)	−14.0 (3.8)	36.5 (5.1)
	*p*	0.25	0.001	**0.001**	**0.001**

^a^ changes from baseline; ^b^ changes from training (i.e., 18 month FU). Bold: effects related to the primary (detraining effects) and secondary (final effects after detraining) hypothesis. CG, control group; HIRT, high-intensity resistance exercise training; LBM, lean body mass; MILES: maximum bilateral isokinetic hip/-leg-extension strength.
